# First report of *Rhodnius montenegrensis* (Hemiptera, Reduviidae, Triatominae) in Amazonas, Brazil

**DOI:** 10.1590/0037-8682-0436-2019

**Published:** 2020-03-16

**Authors:** Fernanda Portela Madeira, André Luiz Rodrigues Menezes, Adila Costa de Jesus, Madson Huilber da Silva Moraes, Jader de Oliveira, João Aristeu da Rosa, Luís Marcelo Aranha Camargo, Dionatas Ulises de Oliveira Meneguetti, Paulo Sérgio Berrnarde

**Affiliations:** 1 Universidade Federal do Acre, Programa de Pós-Graduação Stricto Sensu em Ciências da Saúde na Amazônia Ocidental, Rio Branco, AC, Brasil.; 2 Universidade Federal do Acre, Campus Floresta, Centro Multidisciplinar, Laboratório de Herpetologia, Cruzeiro do Sul, AC, Brasil.; 3 Instituto Federal de Rondônia, Campus de Guajará- Mirim, RO, Brasil.; 4 Universidade Estadual Paulista Júlio de Mesquita Filho, Faculdade de Ciências Farmacêuticas, Departamento de Ciências Biológicas, Araraquara, SP, Brasil.; 5 Universidade Estadual Paulista Júlio de Mesquita Filho, Programa de Pós-Graduação Stricto Sensu em Biociências e Biotecnologia, Araraquara, SP, Brasil.; 6 Universidade de São Paulo, Instituto de Ciências Biomédicas 5, Monte Negro, RO, Brasil.; 7 Centro Universitário São Lucas, Departamento de Medicina, Porto Velho, RO, Brasil.; 8 Centro de Pesquisa em Medicina Tropical de Rondônia, Porto Velho, RO, Brasil.; 9 Instituto Nacional de Epidemiologia da Amazônia Ocidental, Porto Velho, RO, Brasil.; 10 Universidade Federal do Acre, Colégio de Aplicação, Rio Branco, AC, Brasil.

**Keywords:** Western Amazon, Chagas disease, Triatomines

## Abstract

**INTRODUCTION:**

Triatomines are hematophagous insects of epidemiological importance because they are vectors of Chagas disease. We present here the first report of *Rhodnius montenegrensis* in Amazonas, Brazil.

**METHODS:**

Triatomines were collected from *Attalea butyracea* palm trees in the municipality of Guajará.

**RESULTS:**

Two adult female *R. montenegrensis* specimens were identified.

**CONCLUSIONS:**

The present study confirms that the number of triatomine species within the Amazon has increased from 10 to 11, and the number of Brazilian states with *R. montenegrensis* has increased from two to three*.*

Triatomines are hematophagous insects belonging to the Reduviidae family and the Triatominae subfamily^1^. They are found throughout South and Central America and are of epidemiological importance as they are vectors of *Trypanosoma cruzi*, the etiologic agent of Chagas disease (American trypanosomiasis)^1^. These vectors may also transmit the protozoan, *Trypanosoma rangeli*, to vertebrates; although this species does not cause symptoms of infection in humans, it may complicate differential diagnosis of *T. cruzi* ^2^.

Currently, 154 triatomine species, grouped into 19 genera^3^ ^-^ ^5^, have been identified worldwide. Of these, over 30 species, distributed among nine genera, occur within the Amazon region^6^. In the Brazilian state of Amazonas, ten species, distributed among four genera, were previously recorded: *Cavernicola lenti* Barrett and Arias, 1985, *Eratyrus mucronatus* Stal, 1859, *Panstrongylus geniculatus* (Latreille, 1811), *P. lignarius* (Walker, 1873), *P. rufotuberculatus* (Champion, 1899), *Rhodnius amazonicus* Almeida, Santos, and Sposina, 1973, *R. brethesi* Matta, 1919, *R. paraensis* Sherlock, Guitton and Miles, 1977, *R. pictipes* Stal, 1872 and *R. robustus* Larrousse, 1927^7^. The present article reports the first occurrence of *R. montenegrensis* in the Brazilian state of Amazonas.

In January 2019, using a dissection technique, triatomines were collected from four *Attalea butyracea* palm trees (commonly referred to as *Jaci* or *coquinho da mata* in the Amazon region) in a rural area of the municipality of Guajará, Amazonas, near the river Juruá (latitude 07º 30' 87''S, longitude 72º31'17''W). This municipality is located in the meso-region of the southwestern Amazon and the micro-region of Juruá. 

Two triatomines were collected and transferred to the Laboratory of Tropical Medicine at the Federal University of Acre (UFAC) in the city of Rio Branco, Acre, where they were identified based on their morphological characteristics, previously described by Lent and Wygodzinsky^8^ and Rosa et al.^9^. These two specimens were subsequently identified as female *R. montenegrensis* and referred to the Entomology Laboratory of the Department of Biological Sciences, School of Pharmaceutical Sciences, Paulista State University *Júlio de Mesquita Filho* (UNESP), Araraquara, São Paulo, Brazil, where the species were further confirmed by genital charactersistics^10^ (Figure 1A-D). These *R. montenegrensis* individuals (two adult females, municipality of Guajará, Amazonas, Brazil, latitude 07º 30' 87''S, longitude 72º31'17''W, Madeira, F.P, col. Oliveira J, det.) were then added to the Triatominae collection, "Dr Jose Maria Soares Barata" (CTJMSB) of the UNESP, Araraquara.

Collected *R. montenegrensis* individuals were mainly yellow in color with black longitudinal stripes on the pronotum, wings, and connexivum^9^ (Figure 1A). Markings on the head included a distinct central yellow band between two continuous brown bands; stains from the climax regions to the neck were not present^9^ (Figure 1B). Legs were yellow in color, except for the posterior tibias, which had a black stripe near the tarsus^9^(Figure 1A).

**FIGURE 1:**
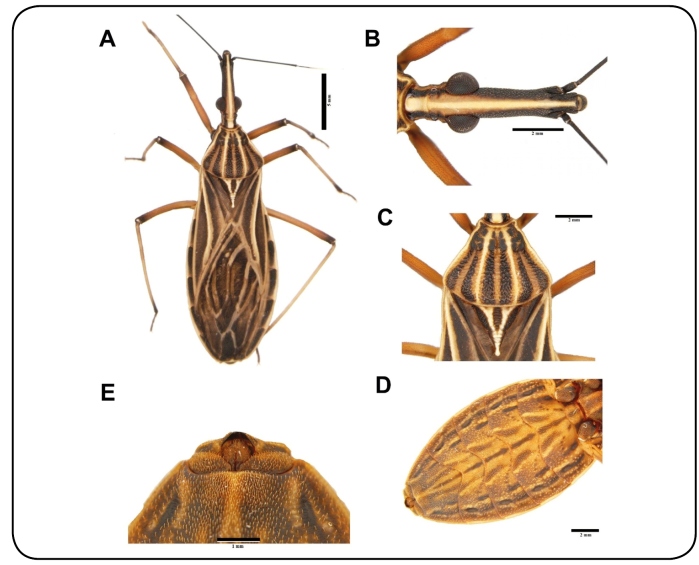
Female specimen of *Rhodnius montenegrensis* collected from *Attalea butyracea* palm trees in the municipality of Guajará, Amazonas, Brazil. **(A)** Dorsal view. **(B)** Head. **(C)** Detail of the pronotum. **(D)** Ventral view. **(E)** Female genitalia.

*R. montenegrensis* specimens were further analyzed for *Trypanosoma sp*. infection. Fresh intestinal content in a 0.9% saline solution was examined using optical microscopy (400x magnification)^11^. *R. montenegrensis* specimens were not positive for trypanosomatids, however.

This is the first report of *R. montenegrensis* in Amazonas. This species was first described in the municipality of Monte Negro, Rondônia^9^, and it was further recorded in two meso-regions in the state of Acre^1^ ^,^ ^12^. Given the results of the present report, the number of triatomines occurring in the state of Amazonas has increased from 10 to 11, expanding the distribution of this species in Brazil, which previously only comprised the states of Rondônia and Acre (Figure 2). 

**FIGURE 2:**
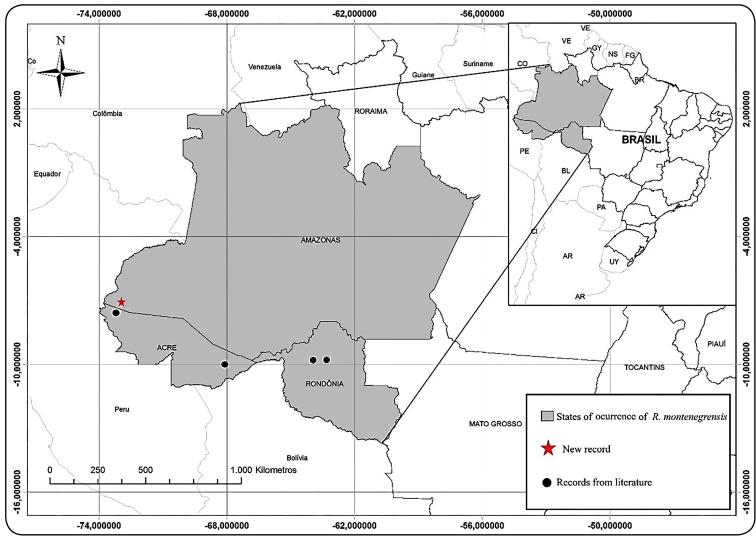
Distribution of *Rhodnius montenegrensis* in Brazil.

Although in the present study, *R. montenegrensis* specimens were not infected by *trypanosomatids*, this species is nonetheless a potential vector for transmission of this etiological agent given that its infection by *T. cruzi* ^13^ and *T. rangeli* ^2^ was previously confirmed. *R. montenegrensis* individuals infected by *T. rangeli* were further found inside apartments in the state of Acre, but without evidence of domiciliation^14^.

The occurrence of *T. cruzi-*infected *R. montenegrensis* individuals indicates that this triatomine has an active role in the maintenance of the enzootic cycle of this trypanosomatid^13^ ^,^ ^15^, thus reinforcing the need for further studies on *R. montenegrensis* occurrence to further elucidate its distribution and overall ecology.
